# Investigating the Sim-to-Real Generalizability of Deep Learning Object Detection Models

**DOI:** 10.3390/jimaging10100259

**Published:** 2024-10-18

**Authors:** Joachim Rüter, Umut Durak, Johann C. Dauer

**Affiliations:** 1 German Aerospace Center (DLR), Institute of Flight Systems, 38108 Braunschweig, Germany; umut.durak@dlr.de (U.D.); johann.dauer@dlr.de (J.C.D.); 2 Institute of Computer Science, Clausthal University of Technology, 38678 Clausthal-Zellerfeld, Germany

**Keywords:** synthetic data, game engine, sim-to-real gap, object detection, deep learning, environment perception, computer vision, air-to-air refueling

## Abstract

State-of-the-art object detection models need large and diverse datasets for training. As these are hard to acquire for many practical applications, training images from simulation environments gain more and more attention. A problem arises as deep learning models trained on simulation images usually have problems generalizing to real-world images shown by a sharp performance drop. Definite reasons and influences for this performance drop are not yet found. While previous work mostly investigated the influence of the data as well as the use of domain adaptation, this work provides a novel perspective by investigating the influence of the object detection model itself. Against this background, first, a corresponding measure called *sim-to-real generalizability* is defined, comprising the capability of an object detection model to generalize from simulation training images to real-world evaluation images. Second, 12 different deep learning-based object detection models are trained and their sim-to-real generalizability is evaluated. The models are trained with a variation of hyperparameters resulting in a total of 144 trained and evaluated versions. The results show a clear influence of the feature extractor and offer further insights and correlations. They open up future research on investigating influences on the sim-to-real generalizability of deep learning-based object detection models as well as on developing feature extractors that have better sim-to-real generalizability capabilities.

## 1. Introduction

Deep learning-based object detection models show steady improvements in accuracy in the recent years. To reach state-of-the-art accuracy, these models rely on huge and diverse datasets. For their practical application in safety-critical domains like aviation, this poses a major problem as it is usually not possible to generate such datasets because of safety and cost reasons. To mitigate the problems of missing real-world datasets, some works investigate the usage of synthetic images generated from game engines. In this work, such images are called simulation images. While this is a promising approach, object detection models trained on simulation images usually have problems generalizing to real-world images ([1,2,3,4,5,6,7,8]). This is shown by a sharp performance drop when the models are applied to real-world images.

Definite reasons and influences on this generalization problem have not yet been found. On an abstract level, the three main components of a deep learning system are *data*, *models*, and *learning* [9]. Therefore, all three components could have influence on the generalization problem. Previous works have mainly focused on the influence of the data. This is a reasonable approach as the data are the input for the training process and differences may be seen with the naked eye. Furthermore, some works have looked into the learning process by investigating different forms of domain adaptation to make the object detection model learn more robust features that could generalize to real-world images (see, e.g., [10,11]). To the best of our knowledge, the influence of the object detection model architecture on the generalizability from simulation to real-world images has not yet been studied. However, we believe that it may also have a significant influence. On the one hand, it is a main component of the deep learning system as described above. On the other hand, different object detection model architectures have been developed in the past years which accomplish certain accuracy improvements. However, it has not been studied whether these improvements also translate to improvements with respect to their generalizability from simulation to real-world images.

Against this background, this paper provides a novel perspective by investigating the generalizability from simulation to real-world image of different deep learning-based object detection models. It is investigated whether the generalizability always aligns with the overall model performance or whether some object detection architectures or feature extractors have less problems generalizing from simulation training images to real-world images. To answer these questions, different deep learning-based object detection models are trained only on simulation images extracted from a game engine and evaluated on real-world images. As object detection models, 12 different combinations of detection architectures and feature extractors are considered and trained with a variation of training hyperparameters resulting in a total of 144 trained versions. As a use-case for this work, the in-air coupling scenario of aerial refueling is chosen because, on the one hand, the dataset appears to be visually simple containing only high-flying aircraft without many reflections and distractions and similar backgrounds without complex textures like tarmac or grass. This allows an isolated investigation of the stated research question as many unknown influences present in more complex datasets can be neglected. On the other hand, the use-case is representative for an object detection system in aviation and therefore may also spur its usage for practical applications. It is worth noting that the European Union Aviation Safety Agency (EASA) even mentions synthetic data in their plans for certifying artificial intelligence components for aviation [12] and therefore, this research has immediate practical relevance. Future work can build on the findings from this use-case and dataset and extend them to more complex use-cases and datasets, e.g., from the automotive domain.

The main contributions of this works are as follows: (i) A definition of the *sim-to-real gap* of a model as well as a definition for *sim-to-real generalizability* are given; (ii) 12 different deep learning-based object detection models are trained on simulation images in a total of 144 training runs using different hyperparameter variations and their sim-to-real generalizability is investigated; (iii) first influences and correlations on the sim-to-real generalizability of deep learning-based object detection models are given. While the general idea of the sim-to-real gap is known in the literature, it lacks a clear definition. Furthermore, different definitions are plausible and discussed in this work. Through these contributions, this work provides a novel perspective on the prevailing challenge of generalizing deep learning object detection models from simulation to real-world images.

## 2. Related Work

Because of the problem of missing real-world data, some work has been conducted in the recent years on using simulation images from game engines for training deep learning models for semantic perception tasks. The first works came from the automotive sector and extracted images for semantic segmentation [1,2,13], object detection [2,14,15], or for more high-level tasks like multi-object tracking [3]. There are also some works using images extracted from game engines for perception tasks from the perspective of an uncrewed aerial vehicle (UAV) like human detection [6,16], vehicle detection [6,7,8,17], animal detection [4,6] or even semantic segmentation [5]. This increasing number of investigations on simulation images is also spurred by a growing amount of high-quality game engines like the one from Grand Theft Auto V [1,14], the UNITY engine [3,13], and the Unreal Engine with its corresponding tools for data extraction like AirSim [18], CARLA [19], and UnrealCV [20] as well as published datasets like *Virtual KITTI* [3], *Synthscapes* [2], *SYNTHIA* [13] as well as the dataset created by Richter et al. [1].

Many works show that using simulation images in combination with real-world ones by training on mixed datasets or using simulation images for pre-training may improve the overall model performance [1,2,3,4,6,13] as well as performance on rare or edge cases [21,22]. It may also help to reduce the number of real-world images needed [1,5,15,23].

However, the described research generally shows that simulation images *alone* are not enough to train a deep learning model that is able to generalize to real-world images but that the models show a significant performance drop when applied to them. This phenomenon is often called *reality gap* or *sim-to-real (domain) gap* and is shown, e.g., in [1,2,3] for semantic segmentation, object detection and multi-object tracking from the car perspective, and in [4,5,6,7,8] for vehicle detection, animal detection, and search and rescue in open water as well as semantic segmentation from the UAV perspective. While some works report a small gap, e.g., for specific use-cases [24], the general trend is unambiguous.

As a result, much work has been conducted to investigate and close the gap. In this context, most work is conducted on the data side, see, e.g., [2,6,8,25,26,27]. On the model side, to the best of our knowledge, not much research has been conducted regarding the influences on the sim-to-real gap. However, it is obvious that some models have better object detection performance than others [28,29]. It may be possible that the same applies for the generalizability from simulation to real-world images. For example, in the context of person detection in artwork, the performance of a YOLO model [30] has been shown to degrade less than other models when trained on real-world images and being applied to artwork [30].

## 3. Investigated Deep Learning Models

In this work, the sim-to-real generalizability of multiple object detection models is investigated. In this section, the used models are described. The selection of the object detection models is based on multiple criteria. Most importantly, the models should be influential or widely used as shown by a high number of citations. All models evaluated have more than 4000 citations according to *Google Scholar* or *Semantic Scholar* as of 11 June 2024. Furthermore, open-source implementations as well as pre-trained weights should be available to reduce potential for errors and to reduce computational load. Because of these criteria, the evaluated models do not include the most recent state-of-the art models. This is justifiable since the main objective is to investigate differences in generalization capabilities from simulation to real-world images between different models and not to investigate absolute achieved detection performance.

Overall, as object detection models *Faster Region Convolutional Neural Network* (Faster R-CNN) [31], *Single Shot MultiBox Detector* (SSD) [32], *RetinaNet* [33], and *Fully Convolutional One-Stage Object Detection* (FCOS) [34] are used. These handle the final object localization and classification. As feature extractors, *Visual Geometry Group* (VGG) models [35], *Residual Neural Networks* (ResNets) [36], *Wide ResNets* [37], *ResNeXt* [38], and *MobileNetV3* [39] models are used. These extract high-dimensional feature maps from the raw input images are used for the subsequent object localization and classification tasks. The following sections briefly describe the used object detection models as well as the used feature extractors.

### 3.1. Feature Extractors

VGG models [35] are a family of convolutional neural networks with a simple architecture of 13, 16 or 19 convolutional layers, called VGG-13, -16, and -19, respectively. ResNets [36] introduce a residual learning framework with residual blocks and shortcut connections. Variants include ResNet-34, -50, -101, differing in their number of layers. ResNet-50 and deeper use a bottleneck design for computational efficiency. Wide ResNets [37] build on ResNets by increasing the width, i.e., number of feature map channels, rather than just depth. They are denoted by WRN-n-k, where *n* is the number of layers and *k* is the widening factor compared to the original ResNet width. ResNeXt [38] extends ResNet by splitting each residual block into *C* branches of width *w*, performing transformations on the feature maps which are aggregated again by element-wise addition. They can be denoted by *ResNeXt-N (C x wd)*, where *N* is the number of layers and *C* and *w* as above. MobileNetV3-Large [39] builds on previous MobileNets [40,41,42] using MnasNet [42] and further network architecture search methods to find an optimal configuration. It comes in MobileNetV3-Small and -Large variants for different computational needs.

### 3.2. Object Detectors

Faster R-CNN [31] is a two-stage detector improving Fast R-CNN [43] with a Region Proposal Network (RPN) for faster region proposals which are used to predict final object classes. SSD [32] is a one-stage detector predicting bounding boxes and class probabilities directly from feature maps, using multiple feature maps of different sizes and default bounding boxes. RetinaNet [33] is a one-stage detector that uses the novel loss function *Focal Loss* to focus on hard samples during training. It combines the feature extractor with a Feature Pyramid Network (FPN) [44] and two subnetworks for class scores and bounding box regression. FCOS [34] is a fully convolutional one-stage detector using per-pixel prediction similar to semantic segmentation. It is anchor box-free, directly regressing bounding boxes at each feature map location. Feature Pyramid Network [44] enhances feature extractors by creating a feature pyramid for multi-scale object detection, and can be integrated into most object detection architectures.

## 4. Evaluation Metrics

In this work, two main evaluation metrics are considered. First, the absolute performance on real-world images when the model is trained only on simulation images is of interest. This measure is of importance for practical applications as it directly quantifies the final performance on the real-world dataset. However, for a model with bad performance on real-world images, this metric does not take into account whether the model learned any useful features at all, i.e., also has bad performance on simulation images, or whether the model is just not able to generalize to real-world images. Therefore, second, a metric to quantify the generalizability to real-world images in reference to the performance on simulation images is used. This metric is useful to investigate different object detection models and model-based influences on the generalization capabilities.

To evaluate the absolute object detection performance of a deep learning object detection model, established metrics exist. This work employs the widely used *mean Average Precision* (mAP) averaged over *Intersection over Union* (IoU) thresholds 0.5, 0.55, ..., 0.95 from the COCO evaluation [45]. In the following, it is denoted mAP(X) when applied on dataset X.

To evaluate the model generalizability to real-world images in reference to the performance on simulation images, we build on the proxy *sim-to-real gap* as defined in the next section. While the general idea of the sim-to-real gap is known in the literature, it lacks a clear definition. Furthermore, different definitions are plausible and discussed below. A machine learning model is then defined to have a good sim-to-real generalizability if it has a low sim-to-real gap.

We let *R* be a dataset containing real-world images and *S* be a dataset containing simulation images. These may be further split into disjoint sets for training $R_{\mathrm{train}}\subset R$, $S_{\mathrm{train}}\subset S$ and testing $R_{\mathrm{test}}\subset R$, $S_{\mathrm{test}}\subset S$. Further, we let $m_{R}$ and $m_{S}$ denote an object detection model trained on $R_{\mathrm{train}}$ and $S_{\mathrm{train}}$, respectively, and let e(m,Y)→[0,100] be an evaluation function for an object detection model *m* on the test set *Y* for $Y\in \{R_{\mathrm{test}},S_{\mathrm{test}}\}$. The sim-to-real gap $g_{\mathrm{abs}}$ may then be defined in two different ways, either by

(1) $$g_{\mathrm{abs}}^{v1}:=e\left(m_{R},R_{\mathrm{test}}\right)-e\left(m_{S},R_{\mathrm{test}}\right)$$

or by

(2) $$g_{\mathrm{abs}}^{v2}:=e\left(m_{S},S_{\mathrm{test}}\right)-e\left(m_{S},R_{\mathrm{test}}\right)$$

where the superscripts v1 and v2 denote the different Variants 1 and 2. Both variants offer a sim-to-real gap in percent points (pp) and have different advantages and disadvantages.

Equation (1) measures how well the model trained on simulation images performs on the real-world images compared to a model trained directly on real-world images. It implicitly takes the complexity of the real-world images into account by incorporating the performance of the model trained directly on the real-world images. Although this formulation is often used in the literature [1,5,6,14], its calculation poses some problems. As *R* usually contains only a very limited amount of images, $m_{S}$ is trained on many more images or one can only use a fraction of the simulation images. This may skew the results. Furthermore, more real-world images are needed for the calculation which are usually not available in this problem setting. On the other hand, Equation (2) does not require any model training on real-world images. This also allows to calculate the sim-to-real gap for one model in isolation. On the downside, the complexity of the real-world images is not taken into account. As we want to investigate the gap for each model, we use Equation (2) as the definition of the sim-to-real gap in the remaining parts of this work, i.e., $g_{\mathrm{abs}}:=g_{\mathrm{abs}}^{v2}$. As a result, a machine learning model with a good sim-to-real generalizability is able to reach a similar performance on real-world images as on simulation images even though it was trained only on simulation images.

Different machine learning object detection models typically show varying levels of performance when evaluated using common object detection metrics. To take this into account, it is also useful to relate it to the overall performance of the model by calculating

(3) $$g_{\mathrm{rel}}:=\frac{g_{\mathrm{abs}}}{e(m_{S},S_{\mathrm{test}})}\cdot 100.$$

Compared to the Definitions (1) and (2), $g_{\mathrm{rel}}$ offers a sim-to-real gap in percents instead of pp.

As evaluation metric *e* in Definitions (1)–(3), this work employs the mAP averaged over *Intersection over Union* (IoU) thresholds 0.5, 0.55, ..., 0.95 from the COCO evaluation [45] as described above.

## 5. Experimental Setup

### 5.1. Dataset

**Figure 1:**
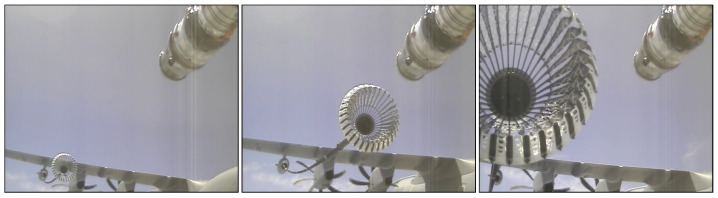
Examples of the available real-world images. Shown is an air-to-air refueling approach from the perspective of the receiving aircraft. Source: [24,46].

To perform the experiments, the dataset from [24] is used. It consists of a real-world dataset as well as a simulation dataset showing images of in-air coupling maneuvers of two aircraft during air-to-air refueling. The real-world dataset is recorded from a camera attached to the probe of the fuel-receiving aircraft and contains 5487 images from five refueling approaches. The images from the shortest refuelling approach are used as a validation dataset $R_{\mathrm{val}}$, containing 518 images. The remaining 4969 images from the other approaches are combined in the test dataset $R_{\mathrm{test}}$. There is no real-world training dataset, as the models are trained only on simulation images. Some example images are shown in Figure 1.

**Figure 2:**
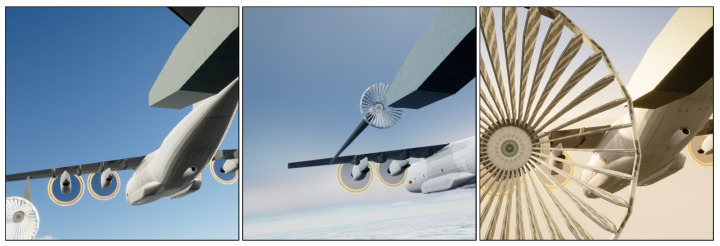
Example simulation images used in this work. Source: [24].

To generate the simulation images, in [24], the use-case of aerial refueling and relevant standard documents were analyzed. From this research, authors derived parameters relevant for a diverse data generation. Additionally, a toolchain to generate images of an aerial refueling situation under different environmental conditions was developed using the Unreal Engine. Using this toolchain and the derived parameters, simulation images were generated. The parameters were selected so that they align with the processes described in the aerial refueling standards and not to exactly represent the available real-world data to not introduce bias. Only the camera parameters like field-of-view and resolution were taken from the real-world camera and the used 3D models were selected to be similar to the aerial objects of the real-world images. For this work, approximately 17,500 simulation images are available. The simulation dataset is split into a training, a validation, and a test dataset called $S_{\mathrm{train}}$, $S_{\mathrm{val}}$ and $S_{\mathrm{test}}$. They contain approximately 60%, 10%, and 30% of the images, respectively. Some example images are shown in Figure 2. Based on the generation process of the simulation images, both datasets visually depict the same scenario as can be seen in Figure 1 and Figure 2. Although there are no exact matching pairs between the simulation and real-world images, they are visually comparable. Visual differences between the simulation and the real-world images can mainly be observed in the rotors of the tanker aircraft as well as in the probe of the refueling aircraft. Furthermore, some differences in photometric characteristics may be observed.

Similar to [24], all images are center cropped to 544 × 544 pixels. Furthermore, they are rescaled to 320 × 320 pixels to improve training and model speed.

### 5.2. Model Configurations and Training Settings

To compare the influence of different feature extractors, a Faster R-CNN object detector combined with different feature extractors is trained. To compare the influence of different architectures, a ResNet-50 feature extractor is integrated and trained with different object detectors. Overall, the following combinations are considered, resulting in a total of 12 object detection models: Faster R-CNN with VGG13, VGG16, and VGG19; Faster R-CNN with MobileNetV3-Large FPN; Faster R-CNN with ResNet-34 FPN, ResNet-50 FPN and ResNet-101 FPN; Faster R-CNN with Wide ResNet-50-2 FPN; Faster R-CNN with ResNeXt-50_32x4d FPN; SSD with ResNet-50; RetinaNet with ResNet-50 FPN; FCOS with ResNet-50 FPN. SSD with ResNet-50 is trained without FPN as SSD uses its own multi-level prediction architecture. All models are implemented in PyTorch [47] and the feature extractors are pre-trained on the ImageNet dataset [48] as provided by PyTorch. Each of these models is trained with different learning rates and optimizers to minimize potential bias of the results because of unsuitable training hyperparameters. Overall, each model is trained using the three different optimizers Adam [49], Stochastic Gradient Decent (SGD) as well as SGD with momentum factor 0.9 and weight decay 0.0005. For each optimizer, four training runs are conducted with the learning rate set to 0.01, 0.001, 0.0001, and 0.00001, respectively. As a result, each of the 12 models is trained 12 times, resulting in 144 trained versions. Table 1 shows all trained model configurations with their number of parameters.

Each model is trained for a maximum of 200 epochs with a training batch size of 16. To reduce computation when the model reaches a local optimum and does not improve anymore, early stopping with a patience of 20 is used. The model performing best on the validation dataset is used for further evaluation.

The training of the models is conducted on $S_{\mathrm{train}}$. As the research question aims to investigate the sim-to-real generalizability in correspondence with the best possible real-world performance of different object detection models trained only on simulation images, the real-world validation dataset $R_{\mathrm{val}}$ is used to make sure that the model that performs best on real-world images is selected. It is important to note that the use of the real-world validation dataset does not influence the training itself and that the model is trained *only* using simulation images.

During training, different standard augmentation strategies are applied. Used augmentations are horizontal flipping, randomly changing brightness and contrast of the image, applying Gaussian and camera sensor noise as well as blurring the image using a randomly sized kernel. These augmentations are applied using the *Albumentations* library [50].

For evaluation, a real-world and a simulation dataset are needed according to Section 4. For this purpose, $R_{\mathrm{test}}$ and $S_{\mathrm{test}}$ are used.

## 6. Evaluation

**Figure A1:**
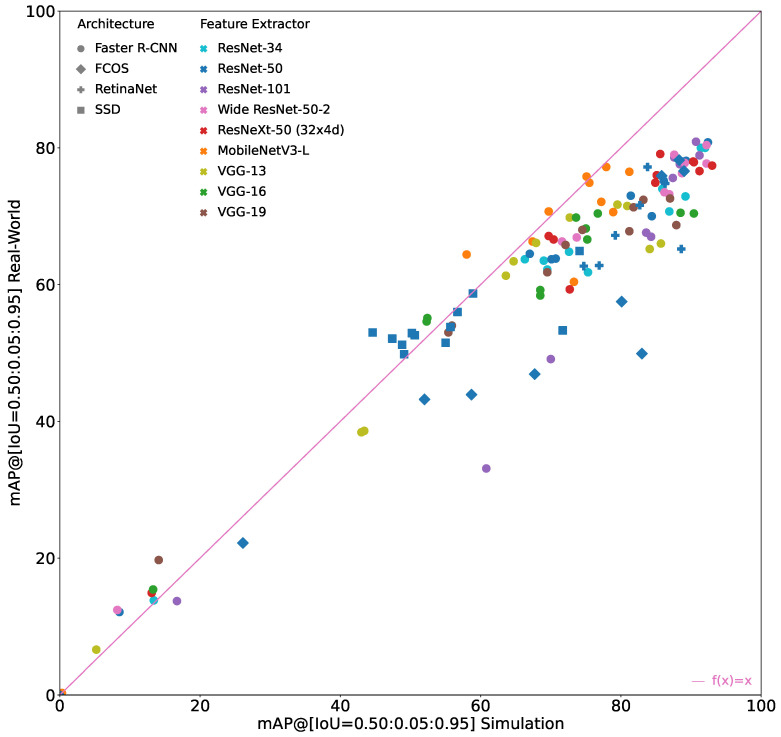
Results of all trained object detection models before removing models that seemingly did not converge with $\mathrm{mAP}(S_{\mathrm{test}})<30\%$ as described in Section 6. X-axis provides $\mathrm{mAP}(S_{\mathrm{test}})$, y-axis provides $\mathrm{mAP}(R_{\mathrm{test}})$. For reference, the identity line is given in pink.

After training, all models with an $\mathrm{mAP}(S_{\mathrm{test}})<30\%$ are removed, as these models do not seem to converge or do not produce meaningful results, indicating a failure to learn effective features under the specific hyperparameter configurations. For the sake of transparency and reproducibility, Figure A1 shows the performance of all trained models before the removal. The focus of the main analysis remains on models with $\mathrm{mAP}(S_{\mathrm{test}})\geq 30\%$. Overall, this results in 115 meaningfully trained models which are evaluated in the next sections.

**Figure 3:**
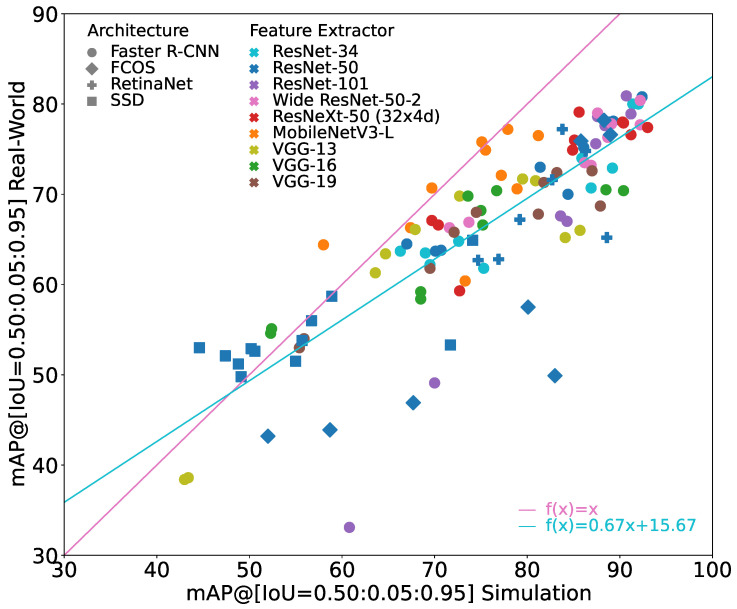
Results of all trained object detection models. X-axis provides $\mathrm{mAP}(S_{\mathrm{test}})$, y-axis provides $\mathrm{mAP}(R_{\mathrm{test}})$. For reference, the identity line is given in pink. The turquoise line indicates the linear regression through all points.

Figure 3 shows all of these models that are considered in the following evaluation.

It can be seen that the sim-to-real generalizability differs significantly between the models. The general trend is that the better the model performs on the simulation images, the better it performs on the real-world images. However, the increase in performance on the real-world images is lower than the increase on the simulation images, i.e., the sim-to-real gap also tends to increase when performance on simulation images increases.

Furthermore, it can be seen that the sim-to-real gap may also be negative, i.e., the performance on the real-world images is better than on the simulation images even though the model was trained only on simulation images. However, this only occurs when the performance on real-world images as well as on the simulation images is relatively low. Therefore, these models are irrelevant for practical applications.

The best overall model in terms of $\mathrm{mAP}(R_{\mathrm{test}})$ is a Faster R-CNN with either a ResNet-50 or a ResNet-101 feature extractor, achieving an $\mathrm{mAP}(S_{\mathrm{test}})$ of 90.7% and an $\mathrm{mAP}(R_{\mathrm{test}})$ of 80.9%. Because of their exact same evaluation results, both models are displayed exactly on top of each other in Figure 3.

### 6.1. Comparison of Different Architectures with ResNet-50 Feature Extractor

**Figure 4:**
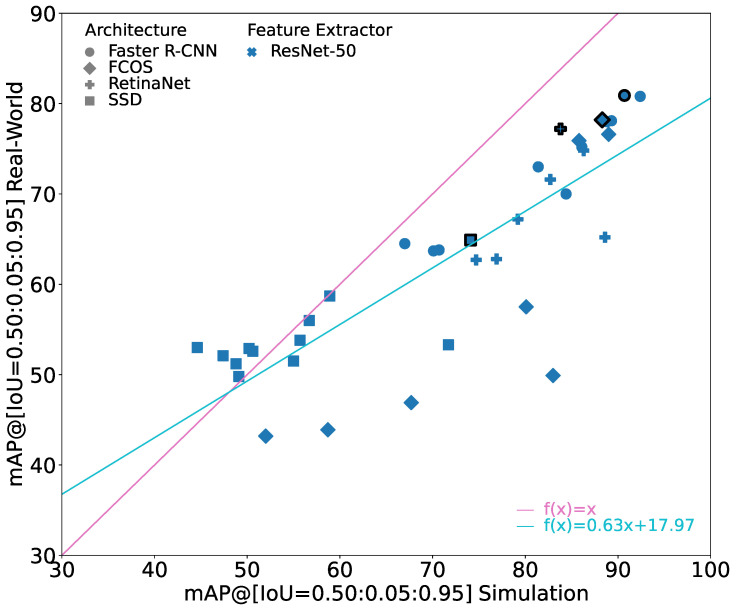
Results of all trained architectures with a ResNet-50 feature extractor. X-axis gives $\mathrm{mAP}(S_{\mathrm{test}})$, y-axis provides $\mathrm{mAP}(R_{\mathrm{test}})$. For reference, the identity line is given in pink. The turquoise line indicates the linear regression through all points. Bold edges highlight the best trained model with respect to $\mathrm{mAP}(R_{\mathrm{test}})$ for each architecture.

Figure 4 shows the performance of the different architectures with a ResNet-50 feature extractor. It shows a subset of Figure 3. The performance of SSD with ResNet-50 on the simulation and the real-world images tends to be relatively low compared to the other models. However, the sim-to-real gap is also relatively small for most of these models. Following the general trend, the gap increases significantly again when looking at the best performing SSD model. FCOS with ResNet-50 seems to be quite sensitive to training parameters, showing comparably low performance on the simulation as well as on the real-world images for some training hyperparameters. Interestingly, compared to the SSD models, the sim-to-real gap is significantly higher for these models. However, when training parameters are fitting, the FCOS models reach a good performance on the simulation images with a sim-to-real gap comparable to the best models. Faster R-CNN and RetinaNet show similar behavior with Faster R-CNN usually showing slightly higher performance on the real-world dataset. Their sim-to-real gap follows the general trend.

In applications in which it is not possible to compare different training hyperparameters, it therefore cannot be recommended to use FCOS as the object detection architecture. In these cases, it seems to be better to use Faster R-CNN as the architecture as it constitutes models that generally have a relatively low sim-to-real gap.

In Figure 4, the marker for the model with the best result on the real-world dataset for each architecture with a ResNet-50 is shown with bold edges. The corresponding values are given in Table 2. When looking only at these best performing models, it can be seen that the architecture has a clear influence on the overall performance of the model with a deviation of more than 15 pp. The sim-to-real gap, however, is relatively similar for all these models and varies only between 6.6 and 10.1 pp, resulting in a deviation of less than 5 pp. In relative terms, the performance on the real-world images is between 7.9% and 12.4% worse than on the simulation images for these models.

Overall, it can be seen that the object detection architecture has an influence on the overall performance of the object detection model. However, while it was discussed above that the object detection architecture has influence on the sim-to-real gap when training hyperparameters are not optimal, it seems to only have limited influence on the overall performance of the object detection model when the hyperparameters are chosen appropriately. Furthermore, while it is often the case that two-stage detection architectures outperform one-stage detection architectures, as is the case in these experiments, the two-stage Faster R-CNN does not show clear advantages in terms of sim-to-real generalizability.

### 6.2. Comparison of Faster R-CNNs with Different Feature Extractors

**Figure 5:**
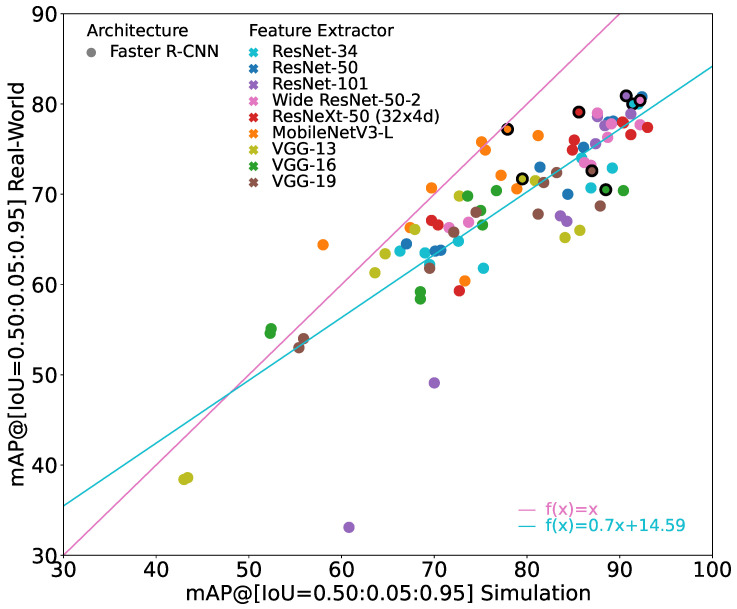
Results of all trained Faster R-CNNs with different feature extractors. X-axis gives $\mathrm{mAP}(S_{\mathrm{test}})$, y-axis provides $\mathrm{mAP}(R_{\mathrm{test}})$. For reference, the identity line is given in pink. The turquoise line indicates the linear regression through all points. Bold edges highlight the best trained model with respect to $\mathrm{mAP}(R_{\mathrm{test}})$ for each feature extractor.

Figure 5 shows the performance of the Faster R-CNN models with different feature extractors. It shows a subset of Figure 3. In general, it can be seen that the models with ResNet feature extractors are able to generate the best performance on real-world images. The models with the VGG feature extractor have a significantly lower performance on real-world images. This is especially true for the models with higher performance on simulation images. As their performance on the simulation images does not deviate that much, they also have a much bigger sim-to-real gap. The MobileNets lie between the ResNets and the VGGs in their performance on real-world images. However, they seem to have the best generalization capabilities as they often outperform all other models with the same performance on simulation images in their performance on real-world images.

In Figure 5, the marker for the model with the best result on the real-world dataset for each feature extractor is shown with bold edges. The corresponding values are given in Table 3. When looking at these best performing models, it can be seen that the best ResNet models have a similar performance that deviates by only 1.8 pp on the real-world images and by 5.2 pp in sim-to-real gap. When not considering ResNeXt-50 (32 × 4d), it reduces even further to just 0.9 pp in performance on the real-world images and 2 pp in sim-to-real gap. With their performance, the ResNets outperform all other models on the real-world images. The ResNets are followed by the best model with a MobileNet feature extractor in terms of performance. While it is not able to reach a performance as high as ResNets by performing 3.7 pp lower on the real-world images, it has a sim-to-real gap of only 0.7 pp. This is by far the lowest sim-to-real gap of the best performing models. Interestingly, the MobileNet has by far the fewest parameters compared to the other investigated models. At the same time, however, this may also be the reason that the model has the worst performance on the simulation images, allowing the model to have a small sim-to-real gap. The worst performances on the real-world images are achieved by the models with the VGG feature extractor. Their performance on real-world images is 8.3 to 10.4 pp worse than the performance of the best performing model. The models with the VGG-16 and VGG-19 feature extractor also have the highest relative sim-to-real gap with values of 20.3% and 16.6%. Only the model with the VGG-13 feature extractor has a sim-to-real gap comparable to the ResNets.

#### 6.2.1. Comparison of Models with ResNet Feature Extractor

**Figure 6:**
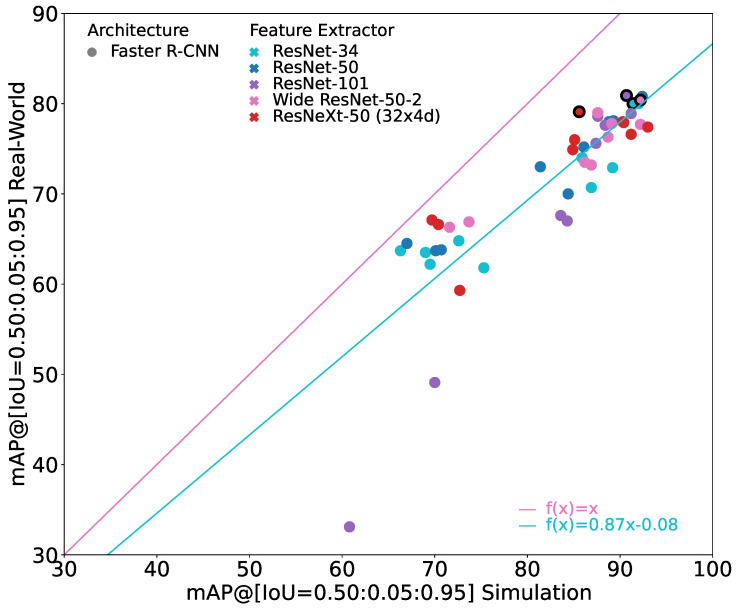
Results of all trained Faster R-CNNs with different ResNet variants as feature extractor. X-axis provides $\mathrm{mAP}(S_{\mathrm{test}})$, y-axis provides $\mathrm{mAP}(R_{\mathrm{test}})$. The identity line is given in pink. The turquoise line indicates the linear regression through all points. Bold edges highlight the best trained model with respect to $\mathrm{mAP}(R_{\mathrm{test}})$ for each feature extractor.

To investigate the influence of the different ResNet feature extractors, Figure 6 shows the results for the ResNet variants in isolation. They show a relatively similar linear-like behavior in terms of performance and sim-to-real gap with only a few exceptions for some models with seemingly suboptimal training hyperparameters. Especially the performance of the best models as highlighted by the bold edges is very similar and deviates only marginally on the real-world images as discussed above.

For the ResNet variants, it can be seen that an increasing number of parameters does not necessarily lead to a better performance or a better sim-to-real generalizability. The models with the Wide ResNet-50-2 feature extractors do not show any improvements in these categories. While the best performing model with a Wide ResNet-50-2 has the best performance on the simulation images, its performance on the real-world images does not improve compared to ResNet-50. Neither is the case for an increasing number of layers as seen for ResNet-34, -50 and -101. The contrary seems to be the case as ResNet-101 even seems to be more sensitive to training hyperparameters, leading to models with low performance and a large sim-to-real gap as seen in the lower half of Figure 6. Interestingly, the models with ResNet-50 and ResNet-101 feature extractors show similar performance, even though ResNet-101 has more than twice the number of layers and more than 20,000 additional parameters. The residual connections of ResNets allow the training of this deep network but also allow multiple layers to be skipped during training. It may therefore be possible that the capabilities of ResNet-101 are not needed or exploited entirely. This is in line with the common problem that deeper machine learning models are usually harder to train than shallow ones. The introduced *cardinality* dimension of the ResNeXt models also does not show clear signs of improving the sim-to-real gap. Most of the trained models lie in the proximity of the other models with ResNet feature extractors. While its sim-to-real gap is the lowest of the best performing models with ResNet feature extractors, its overall performance on the real-world images is slightly worse.

#### 6.2.2. Comparison of Models with VGG Feature Extractor

**Figure 7:**
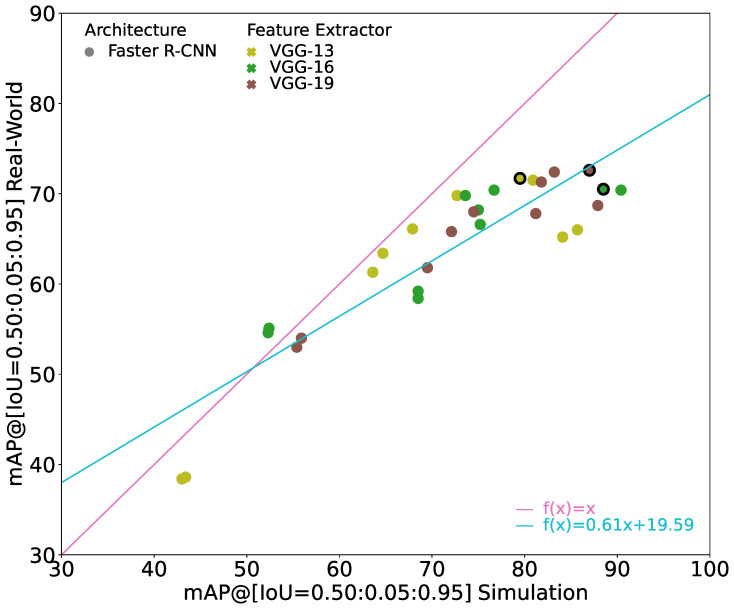
Results of all trained Faster R-CNNs with different VGG variants as feature extractor. X-axis provides $\mathrm{mAP}(S_{\mathrm{test}})$, y-axis provides $\mathrm{mAP}(R_{\mathrm{test}})$. The identity line is given in pink. The turquoise line indicates the linear regression through all points. Bold edges highlight the best trained model with respect to $\mathrm{mAP}(R_{\mathrm{test}})$ for each feature extractor.

To investigate the influence of the different VGG feature extractors, Figure 7 shows the results for the VGG variants in isolation. Compared to the relatively linear slope of the ResNet models, the slope of the models with VGG feature extractors looks much more like a logarithmic function, i.e., the sim-to-real gap becomes much worse when the performance on simulation increases. Also, the slope of the linear regression is much smaller with 0.61 compared to 0.87. When comparing VGG models with similar performance on simulation images, VGG-13 usually shows better performance on real-world images than VGG-16 and VGG-19, i.e., it has a lower sim-to-real gap. Interestingly, the best models with VGG-13 and VGG-19 feature extractors differ only slightly, by 0.9 pp, in performance on real-world images but by 7.5 pp in performance on simulation images resulting in a larger sim-to-real gap as shown in Table 3. Therefore, it can be said that VGG-13 with less parameters may learn a more robust set of features that is less prone to overfitting with respect to sim-to-real generalizability. However, increasing the number of layers and consequently the number of parameters from VGG-16 to VGG-19 does not show any further degrading behavior.

#### 6.2.3. Influence of Optimizer

The optimal training hyperparameters usually depend on the training data as well as the model to train. Therefore, an evaluation of the influence of the training hyperparameters is not easy to perform. However, it is interesting to note that all of the three best models trained using SGD as optimizer shown in Table 3 have a sim-to-real gap of less than 10 pp.

To investigate this influence of the optimizer further, Table 4 shows statistical values for the best performing model for each of the nine feature extractors when trained with SGD compared to Adam as optimizer. When comparing the performance on real-world images, their upper bounds are the same with 80.9%. However, their lower bounds differ from 66.1% for Adam to 70.1% for SGD. On average, the performance on real-world images differs by only 0.3 pp, while their sim-to-real gap differs by 1.7 pp. To a large part, this can be attributed to a higher maximal sim-to-real gap which reaches 18 pp for Adam compared to only 11.9 pp for SGD. However, the smallest sim-to-real gap is lower for Adam, with only 0.7% compared to 4.7% for SGD.

In the literature, there are some works indicating that Adam can lead to more overfitting compared to SGD (see, e.g., [51,52]). This work shows that while the Adam optimizer does not necessarily lead to models with bad performance on the real-world images as many of the best models on the real-world images use Adam as the optimizer, SGD seems to produce models with a lower maximal sim-to-real gap.

### 6.3. Comparison of Influence of Architecture with Influence of Feature Extractors

When looking at the best performing model for each architecture and feature extractor combination, the experiments show that while the architecture has a larger influence on the overall performance of the object detection model, the feature extractor has a much larger influence on the sim-to-real gap. The sim-to-real gap for the best models with different feature extractors from Table 3 deviates from 0.7 to 18.0 pp, resulting in a bandwidth of 17.3 pp, compared to deviations from 6.6 to 10.1 pp, which yield a bandwidth of 3.5 pp for the different architectures with ResNet-50 feature extractor from Table 2.

The sim-to-real gaps of the different feature extractors also depend much more on the performance on simulation images. While the performance of the different architectures with ResNet-50 feature extractor on the real-world images was 7.9% to 12.4% worse than on the simulation images, the performance of the different feature extractors with Faster R-CNN object detection architecture was 0.9% to 20.3% worse.

## 7. Conclusions and Future Work

This paper investigated the object detection model capability of *sim-to-real generalizability* which describes the ability to generalize from simulation training images to real-world evaluation images. By providing a detailed empirical study on the influence of the object detection architecture as well as the feature extractor on the sim-to-real generalizability, this work offers a novel perspective on the prevailing challenge of generalizing deep learning object detection models from simulation to real-world images. To achieve this, 12 different object detection models were trained only on simulation images and evaluated on simulation images as well as real-world images. The models were trained with a variation of hyperparameters resulting in a total of 144 trained versions. The results highlightedthat object detection models differ significantly in their sim-to-real generalizability capabilities. The general trend was that the better a model performs on the simulation images, the better it performs on the real-world images, but also that the sim-to-real gap tends to increase when performance on simulation images increases. It was also shown that the sim-to-real gap can be negative, meaning models trained on simulation images perform better on real-world images than on simulation evaluation images. However, this only happens when performance on both is relatively low, making these models unsuitable for practical use. As a result of these findings, future investigations on the sim-to-real generalizability, e.g., on the data side should always try to investigate multiple models to average out model-specific influences. While the definite reasons still remain unknown, this work showed first influences and correlations.

Out of the evaluated models, a Faster R-CNN with either a ResNet-50 or a ResNet-101 feature extractor achieved the best performance on real-world images. It was shown that the architecture does not have a huge influence on the sim-to-real gap while the feature extractor does. Therefore, in future work, focus should be shifted on developing better feature extractors. As the sim-to-real generalizability seems to depend not only on their overall performance, future work should also pay attention to improving their sim-to-real generalizability.

Future work should also investigate the influences and correlations from different object detection models on their sim-to-real generalizability further. As it was shown in this paper, that different deep learning-based object detection models differ in their sim-to-real generalizability, it would also be interesting to investigate more state-of-the-art models including transformer architectures. It could also be interesting to evaluate further whether the sim-to-real generalizability improves over time. To achieve this, a different version, e.g., from the YOLO family could be investigated. The results of this work could furthermore be verified on different datasets from different domains and more influencing factors, e.g., from the data side could be investigated. Ultimately, this could allow the usage of deep learning-based object detection models in more domains in which real-world data are hard to acquire.

## Figures and Tables

**Table 1:** List of all the trained model configurations in combination with their number of parameters.

Architecture	Feature Extractor	Number of Parameters
Faster R-CNN	VGG-13	38,554,261
Faster R-CNN	VGG-16	43,863,957
Faster R-CNN	VGG-19	49,173,653
Faster R-CNN	MobileNetV3-Large FPN	18,930,229
Faster R-CNN	ResNet-34 FPN	38,374,617
Faster R-CNN	ResNet-50 FPN	41,299,161
Faster R-CNN	ResNet-101 FPN	60,239,065
Faster R-CNN	WRN-50-2 FPN	84,610,265
Faster R-CNN	ResNeXt-50 (32x4d) FPN	40,755,929
SSD	ResNet-50	25,167,002
RetinaNet	ResNet-50 FPN	32,168,694
FCOS	ResNet-50 FPN	32,064,455

**Table 2:** Comparison of different architectures with ResNet-50 feature extractor. Shown are the models with the highest mAP($R_{\mathrm{test}}$) for each combination. LR denotes learning rate, Optim denotes optimizer, and WD denotes weight decay. Values for mAP($S_{\mathrm{test}}$), mAP($R_{\mathrm{test}}$) and $g_{\mathrm{rel}}$ are given in percents, values for $g_{\mathrm{abs}}$ are given in percentage points. Best values for each column are marked in bold, worst values are marked in cursive.

Architecture	LR	Optim	WD	mAP (*S*_test_)	mAP (*R*_test_)	*g*_abs_	*g*_rel_
Faster R-CNN	1.0 $\times \;10^{-4}$	Adam	No	**90.7**	**80.9**	9.8	10.8
FCOS	1.0 $\times \;10^{-4}$	Adam	No	88.3	78.2	*10.1*	11.4
RetinaNet	1.0 $\times \;10^{-5}$	Adam	No	83.8	77.2	**6.6**	**7.9**
SSD	1.0 $\times \;10^{-4}$	Adam	No	*74.1*	*64.9*	9.2	*12.4*

**Table 3:** Comparison of Faster R-CNNs with different feature extractors. LR denotes learning rate, Optim denotes optimizer and WD denotes weight decay. Values for mAP($S_{\mathrm{test}}$), mAP($R_{\mathrm{test}}$) and $g_{\mathrm{rel}}$ are given in percents, values for $g_{\mathrm{abs}}$ are given in percentage points. Best values for each these column are marked in bold, worst values are marked in cursive.

Feature Extractor	LR	Optim	WD	mAP (*S*_test_)	mAP (*R*_test_)	*g*_abs_	*g*_rel_
ResNet-101	1.0 $\times \;10^{-3}$	SGD	Yes	90.7	**80.9**	9.8	10.8
ResNet-50	1.0 $\times \;10^{-4}$	Adam	No	90.7	**80.9**	9.8	10.8
Wide ResNet-50-2	1.0 $\times \;10^{-4}$	Adam	No	**92.2**	80.4	11.8	12.8
ResNet-34	1.0 $\times \;10^{-4}$	Adam	No	91.4	80.0	11.4	12.5
ResNeXt-50 (32x4d)	1.0 $\times \;10^{-2}$	SGD	Yes	85.6	79.1	6.5	7.6
MobileNetV3-L	1.0 $\times \;10^{-5}$	Adam	No	*77.9*	77.2	**0.7**	**0.9**
VGG-19	1.0 $\times \;10^{-4}$	Adam	No	87.0	72.6	14.4	16.6
VGG-13	1.0 $\times \;10^{-2}$	SGD	No	79.5	71.7	7.8	9.8
VGG-16	1.0 $\times \;10^{-5}$	Adam	No	88.5	*70.5*	*18.0*	*20.3*

**Table 4:** Comparison of statistical values of the best performing Faster R-CNNs with respect to mAP $R_{\mathrm{test}}$ for each of the nine feature extractors when trained with Adam compared to SGD optimizer. Values for mAP $R_{\mathrm{test}}$ are given in percents and values for $g_{\mathrm{abs}}$ are given in percentage points. Best values for each row are marked in bold. Given are Mean, Standard Deviation (Std), Minimum (Min), and Maximum (Max) Values.

	mAP $R_{\mathrm{test}}$		*g*_abs_	
	Adam	SGD	Adam	SGD
Mean	**76.1**	75.8	10.3	**8.6**
Std	**5.2**	3.8	5.6	**2.5**
Min	66.1	**70.4**	**0.7**	4.7
Max	**80.9**	**80.9**	18.0	**11.9**

## Data Availability

The simulation dataset was kindly provided by the authors of [24]. The real-world images were kindly provided by Wehrtechnische Dienststelle für Luftfahrzeuge und Luftfahrtgerät der Bundeswehr (WTD 61) for research purposes.

## Abbreviations

The following abbreviations are used in this manuscript:

IoU	Intersection Over Union
mAP	Mean Average Precision
pp	Percent Points
SGD	Stochastic Gradient Decent
